# 299 Donor Site Reduction with Combined Use of Bromelain Enzymatic Debridement and Autologous Skin Cell Suspension

**DOI:** 10.1093/jbcr/irad045.274

**Published:** 2023-08-29

**Authors:** Steven A Kahn, Jeffrey E Carter, Gabriel G Gaweda, Elizabeth Halicki, William L Hickerson, Ashley Hink, James H Holmes, Deepak Ozhathil, Narayan Raghava

**Affiliations:** The South Carolina Burn Center/Medical University of South Carolina, Charleston, South Carolina; University Medical Center New Orleans & LSUHSC, River Ridge, Louisiana; MUSC, Charleston, South Carolina; South Carolina Burn Center @ MUSC, Charleston, South Carolina; LSUHSC, Memphis, Tennessee; Medical University of South Carolina, Charleston, South Carolina; WFSOM, Winston-Salem, North Carolina; South Carolina Burn Center at MUSC, Charleston, South Carolina; MUSC, Charleston, South Carolina

## Abstract

**Introduction:**

The conventional treatment for deep 2^nd^ and 3^rd^ degree burn injuries has been excision and grafting, which was first described approximately 80 years ago. New techniques and technology in the field of burn surgery that facilitate “minimally invasive” donor site reduction include bromelain enzymatic debridement and autologous skin cell sprays (ASCS). Each of these techniques independently have been shown to decrease the need of autografting and reduce the donor site size. However, the use of both methods together was not possible until recently. The authors hypothesize that bromelain enzymatic debridement plus ASCS will result in donor site reduction compared to conventional excision and grafting.

**Methods:**

A single institution retrospective review of patients who underwent treatment of deep 2^nd^ and 3^rd^ degree burns was performed. The patients were stratified into those that received conventional excision and autografting (CONTROL group), and those that received bromelain enzymatic debridement with autologous skin cell spray with or without a split thickness skin graft (TREATMENT group). Data collection included demographics, injury characteristics, length of stay (LOS)/%TBSA. Total surface area treated with ASCS, autograft, and donor site size was tabulated. A ratio of donor site to total area treated was calculated and compared to determine relative reduction in donor site size. Data was reported in medians+IQR and comparisons were done with a Mann Whitney test.

**Results:**

Twenty-eight study patients were included over 18 months, 15 CONTROLS and 13 TREATMENT patients. Burns size was 1789 cm^2^ (IQR: 450, 2434) and 1463cm^2^ (IQR: 699, 2740.5) in the CONTROLS and TREATMENT groups, respectively. There were no differences in age and sex. The total area grafted and/or treated with ASCS was 1342 cm^2^ (IQR: 824, 2535) in the CONTROLS and 1404 cm^2^ (IQR: 1021.5, 2430.5) in the TREATMENT group (p=0.4354). The donor site area to total treatment area ratio for CONTROLS was 0.273 (IQR:0.18,0.47) while the TREATMENT group was 0.082 (IQR:0.039,0.241) (p=0.013). The O/E ratio for LOS was 1.35 days/%TBSA for the CONTROLS and 1.06 for the TREATMENT group (p= 0.2). No significantly differences were detected in comorbidities between groups. Each group had 1 unplanned readmission but no graft loss. There were no mortalities.

**Conclusions:**

Bromelain based enzymatic debridement can be successfully utilized with ASCS to perform a “minimally invasive” skin graft. These patients received relatively smaller donor sites and showed a trend for lower LOS. Just as with all surgical therapies, appropriate patient selection and burn characteristics are imperative when utilizing minimally invasive burn surgery. Further larger scale studies are needed to adjust for confounders and determine the individual and synergistic effects of the component novel therapies.

**Applicability of Research to Practice:**

Donor site reduction improves care paradigms

